# A metabolomic endotype of bioenergetic dysfunction predicts mortality in critically ill patients with acute respiratory failure

**DOI:** 10.1038/s41598-021-89716-0

**Published:** 2021-05-18

**Authors:** Raymond J. Langley, Marie E. Migaud, Lori Flores, J. Will Thompson, Elizabeth A. Kean, Murphy M. Mostellar, Matthew Mowry, Patrick Luckett, Lina D. Purcell, James Lovato, Sheetal Gandotra, Ryan Benton, D. Clark Files, Kevin S. Harrod, Mark N. Gillespie, Peter E. Morris

**Affiliations:** 1 University of South Alabama College of Medicine, Mobile, AL USA; 2 Wake Forest Baptist Medical Center, Winston-Salem, NC USA; 3 Duke University Center for Genomic and Computational Biology, Durham, NC USA; 4 Washington University in Saint Louis, Saint Louis, MO USA; 5 University of South Alabama School of Computing, Mobile, AL USA; 6 University of Alabama-Birmingham College of Medicine, Birmingham, AL USA; 7 Division of Pulmonary, Critical Care and Sleep Medicine, University of Kentucky Health Care, 206E Mathews Building, Lexington, KY 40506-0047 USA

**Keywords:** Biochemistry, Systems biology, Biomarkers, Medical research

## Abstract

Acute respiratory failure (ARF) requiring mechanical ventilation, a complicating factor in sepsis and other disorders, is associated with high morbidity and mortality. Despite its severity and prevalence, treatment options are limited. In light of accumulating evidence that mitochondrial abnormalities are common in ARF, here we applied broad spectrum quantitative and semiquantitative metabolomic analyses of serum from ARF patients to detect bioenergetic dysfunction and determine its association with survival. Plasma samples from surviving and non-surviving patients (N = 15/group) were taken at day 1 and day 3 after admission to the medical intensive care unit and, in survivors, at hospital discharge. Significant differences between survivors and non-survivors (ANOVA, 5% FDR) include bioenergetically relevant intermediates of redox cofactors nicotinamide adenine dinucleotide (NAD) and NAD phosphate (NADP), increased acyl-carnitines, bile acids, and decreased acyl-glycerophosphocholines. Many metabolites associated with poor outcomes are substrates of NAD(P)-dependent enzymatic processes, while alterations in NAD cofactors rely on bioavailability of dietary B-vitamins thiamine, riboflavin and pyridoxine. Changes in the efficiency of the nicotinamide-derived cofactors’ biosynthetic pathways also associate with alterations in glutathione-dependent drug metabolism characterized by substantial differences observed in the acetaminophen metabolome. Based on these findings, a four-feature model developed with semi-quantitative and quantitative metabolomic results predicted patient outcomes with high accuracy (AUROC = 0.91). Collectively, this metabolomic endotype points to a close association between mitochondrial and bioenergetic dysfunction and mortality in human ARF, thus pointing to new pharmacologic targets to reduce mortality in this condition.

## Introduction

Acute respiratory failure (ARF) is a challenging clinical problem with ~ 180,000 cases nationally and an in-hospital mortality of 38.5%^1^. Several potential biomarkers have been identified; however, many of the study results are inconsistent due to differences in sampling methods or are insignificant due to low sample numbers^2^. Although neither biomarkers to predict evolution of ARF nor pharmacologic strategies to forestall its progression are currently available, a series of reports point to the prospect that mitochondrial (mt)-associated pathways—frequently disrupted in critical illness in both animal models and human patients—could be responsive to both unmet needs^3–9^.

Metabolomics is emerging as a powerful approach to identify disease biomarkers. In one of its earliest applications in sepsis, we evaluated a pool of about 300 serum metabolites and developed a composite metabolomic biomarker that accurately predicted outcomes in septic patients on admission to the emergency department (ED) or to the medical intensive care unit (MICU)^8,10^. To delineate mechanistic links between metabolomic abnormalities and sepsis outcomes, we next executed a study in a non-human primate (NHP) model of sepsis, combining serum metabolomics and transcriptomics derived from intact lung tissue. Here, we validated many of the metabolomic changes noted in septic human patients and were able to identify four distinct biochemical pathways that were related to sepsis diagnosis and outcomes^9^: (1) Decreased acyl-glycerophosphocholines (-GPCs) which appear linked to platelet activating factors (PAF) and to increased reactive oxygen species (ROS)-mediated bacterial killing in neutrophils^11,12^; (2) Increased taurine-conjugated bile acids that are predictive of liver cholestasis^13^; (3) Increased kynurenine pathway-associated metabolites which are related to dysregulated endogenous nicotinamide adenine dinucleotide (NAD) biosynthesis^14^, and; (4) Increases in small- and medium-chain fatty acids and branched-chain amino acids (BCAA) bound to carnitine which we subsequently refer to as “acylcarnitines”^8–10,15^.

Sepsis and ARF, a severe critical illness often due to sepsis, are heterogeneous disorders which can be complicated by the site of infection, infection source, timing and appropriateness of therapeutic interventions, comorbidities, age, and genetic predispositions^16,17^. These heterogeneous pathophysiologic changes are often difficult to recapitulate in animal models^17,18^. However, the molecular and metabolomic changes found in humans and the NHP model of sepsis suggest a bioenergetic crisis leading to poor outcomes. Therefore, we hypothesized we could identify an endotype related to ARF; in other words, a distinct pathophysiologic or functional subtype that can both differentiate risk of disease as well as potential response to therapy^19^. This could not only lead to development of a new predictive biomarker but also point to new pharmacologic targets for intervention. Accordingly, the proximate goal of the present study was to determine the metabolomic changes related to ARF as well as develop a biomarker-based model that should uncover a metabolomic endotype that potentially guides emerging pharmacologic or nutritional strategies that reverse mitochondrial dysfunction^20^, immunosuppression^21^, or muscle wasting^22^.

To test this hypothesis, semiquantitative and quantitative ultrahigh performance liquid chromatography mass spectrometry (UHPLC MS) analysis was performed on patient serum collected from the **T**rial with **A**cute **R**espiratory failure patients: evaluation of **G**lobal **E**xercise **T**herapies; TARGET^23^. Extensive pathway analysis of the biochemical changes was performed and a **Met**abolomic **Se**psis Outcomes **P**rediction (MetSeP) score system was developed to determine whether the disruption in specific metabolic pathways can identify the bioenergetic and metabolomic profile of these patients.

## Methods

This study is a retrospective analysis of patients that were enrolled in a single center, randomized clinical trial at Wake Forest Baptist Medical Center, North Carolina (TARGET**;** ClinicalTrials.gov Identifier: NCT00976833)^23^. The study was approved by the Wake Forest Baptist Medical Center institutional review board, and informed, written consent was obtained from the study participants or an authorized legal representative. All experimental protocols were approved by Wake Forest Baptist Medical Center, and the methods performed were in accordance with the relevant guidelines and regulations. Inclusion and exclusion criteria were previously described^23^. Briefly, adults (≥ 18 years) admitted to the MICU requiring mechanical ventilation by endotracheal tube or noninvasive ventilation by mask and an arterial oxygen partial pressure to fractional inspired oxygen (PaO_2_/FIO_2_) ratio < 300 mmHg were included. Patients were excluded due to inability to walk without assistance, cognitive impairment prior to admission, body mass index > 50, neuromuscular disease, unstable cervical spine or pathologic fracture, mechanical ventilation more than 80 h, current hospitalization more than 7 days, do not intubate designation on admission, considered to have moribund status by the primary attending physician, or if they were enrolled in another research study. For this study, patients were further excluded if they had diagnosed cirrhosis or chronic renal failure that required hemodialysis as these conditions can affect metabolomic profiles^8^. Enrollment followed a Convenience sampling approach. For this nested-case control study, survivors (> 180d post enrollment) were matched by age, race, sex, and randomized control trial (RCT) grouping to the corresponding nonsurvivors (< 28-day mortality post enrollment; Table 1). Serum was sampled in survivors (n = 15) and nonsurvivors (n = 15) at enrollment (day 1), day 3, and hospital discharge in survivors.

**Table 1:** Patient demographics.

	Death	Survival	*P* values
	N = 15	N = 15	
Age, mean (CI)	52.4 (43.4–61.4)	52.9 (43.4–61.3)	0.8
Sex			
Female (%)	4 (26.7)	4 (26.7)	1
Male (%)	11 (73.3)	11 (73.3)	
Race/Ethnicity			
White (%)	10 (66.7)	9 (60)	0.7
Black/African American (%)	5 (33.3)	6 (40)	
APACHE III score, mean (CI)	96.9 (82.1–111.8)	71.8 (51.3–92.3)	0.016
Lactate, Mean (CI)^a^	4.6 (2.3–6.9)	2.2 (1.2–3.2)	0.115
Intensive care unit diagnosis			
Coma (%)	0 (0)	1 (6.7)	0.59
Acute respiratory failure			
Without chronic lung disease (%)	12 (80)	11 (73.3)	
With chronic lung disease (%)	3 (20)	3 (20)	
Home oxygen (%)	0 (0)	3 (20)	0.07
Days from enrollment to death, (CI)	8.3 (4.2–12.3)	N/A	
Days from enrollment to discharge, (CI)	N/A	12.9 (8.3–17.6))	
RCT Assignment			
Intervention (%)	8 (53.3)	8 (53.3)	
Control (%)	7 (46.7)	7 (46.7)	1

*CI* 95% confidence interval.

^a^Survivor n = 9; nonsurvivor n = 12.

### Semiquantitative metabolomic analysis

Metabolon Inc, (Durham, NC) performed broad-spectrum mass spectrometry analysis of patient serum samples as previously described^8–10,15,24^. Briefly, extraction was performed as previously described using 450 μl of methanol to 100 μl of each sample, and four separate aliquots were dried under nitrogen overnight. Two aliquots were reconstituted in 50 μl of 6.5 mM ammonium bicarbonate or 50 μl of 0.1% formic acid in water. Both aliquots included internal instrument standards for LC retention index and evaluating LC/MS instrument performance. A third 110 μl aliquot was derivatized by treatment with 50 μl mixture of N,O-bistrimethylytriflouroacetamide and 1% trimethylchlorosilane cyclohexane/dichloromethane/acetonitrile (5:4:1 ratio) plus 5% tiethylamine and internal standards for GC retention index. The samples were analyzed on a UPLC-Orbi-Elite Instrument (Thermo Fisher Scientific, Waltham, MA, USA) or Trace GC Ultra Gas Chromatograph-Dual Stage Quadrapole GC/MS system (Thermo Fisher Scientific). For each biological matrix, relative standard deviations of peak area were calculated for each internal standard to confirm performance. Peak detection and integration utilized in-house software. The output generates a list of *m/z* ratios, retention times, and area-under-the-curve (AUC) values. Values are normalized in terms of raw area counts. Any metabolites with > 50% of the values missing are removed prior to data analysis. Each biochemical is rescaled to the median equal to one, and missing values are imputed with the minimum.

### Quantitative metabolomic analysis

The Duke Proteomics and Metabolomics Shared Resource (Durham, NC) performed targeted metabolomic analysis using two metabolite quantification kits from Biocrates AG (Innsbruck, Austria), the AbsoluteIDQ p180 and Bile Acids quantification kits. These kits include four metabolomic biomarkers that comprise the MetSeP score (acetylcarnitine, kynurenine, 1-archidonoyl-GPC and taurolithocholic acid sulfate (TLCAS). When coupled to an Acquity UPLC chromatography (Waters Corporation, Milford, MA, USA) and a Xevo TQ-S Triple Quadrupole mass spectrometry (Waters Corporation) delivered quantitative analysis of all required analytes plus approximately 150 others spanning similar metabolite classes (carnitines, amino acids, lipids, and bile acids). The analysis utilizes 10 μl serum for each kit. The p180 kit utilizes a combination of flow-injection analysis selected-reaction monitoring (FIA-SRM) and liquid chromatography SRM (LC-SRM), and the bile acids analysis also uses LC-SRM. The entire sample preparation, data collection, and data analysis are performed based on standard operating procedures (SOP) provided by Biocrates, Inc. The data collection includes calibration curves (8 levels) and QC standards (3 levels). The Duke Core protocol includes two additional control pools: First, a “study pool” which is a pool of all samples analyzed within the study (or representative subsampling); second, a “global reference pool” is analyzed on every kit plate, serving as a reference standard within study.

Raw data is directly imported into the MetIDQ software (Biocrates) for calibration based on the stable-isotope dilution approach against class-based internal standards for each lipid class, and molecule-specific standards for the acylcarnitines. For amino acids, biogenic amines, and bile acids, LC separation enables high specificity and sensitivity. This data is collected by retention-time scheduled Selected Reaction Monitoring. The chromatographic raw data is quantified against standard curves in TargetLynx software (Waters Corporation) and this quantified data is imported into MetIDQ software for tracking and data analysis. The kit demonstrates excellent inter- and intraday precision and accuracy and exhibited excellent inter-day reproducibility across all analyte classes, with 8.7% CV on average for bile acids and 3.0% CV on average for p180 platforms, using the Study Pool QC samples. The targeted metabolomics data has been made available in supplemental Tables 1–4. Any metabolites with > 50% of the values missing are removed, and missing values are imputed with the minimum prior to data analysis.

### Statistical analysis

Analysis of variance (ANOVA), Spearman’s Rank correlation analysis and logistic regression analysis of clinical, semiquantitative and quantitative data was performed using JMP Genomics 8.0 (SAS Inc., Cary NC) as previously described^8,9^. Briefly, raw data provided by Metabolon and Duke, was log2(*x* + 1) transformed and ANOVA with 5% false discovery rate (FDR) was performed. Spearman’s Rank correlation was performed to compare semiquantitative versus quantitative data using JMP Genomics. Logistic regression analysis was performed based off four markers that make up the MetSeP score and presented as area under the receiver-operator curves (AUROC). Bar charts, nonparametric Mann–Whitney tests and 95% confidence intervals (CI) for clinical variables (age, lactate, APACHEIII) were determined using GraphPad Prism 7.0 (GraphPad Software Inc., La Jolla, CA).

## Results

Three hundred patients admitted to the WFBMC were enrolled in the TARGET cohort^23^. This single center, single blind, randomized control study evaluated long term physical function after the initiation of physical therapy versus usual care in patients with ARF. Patients were enrolled into the study within 80 h of initiation of ventilation by mask or endotracheal tube. For this nested case–control study, the first 15 nonsurvivors that died within 28d-post enrollment were selected. We also selected 15 survivors (180d survival post enrollment) that matched nonsurvivors for age, race, gender, and RCT grouping (Table 1).

The time from patient discharge in survivors was 12.8 days (CI 8.3–17.8), while time to death was 8.3 days (CI 4.2–12.3). Lactate values were measured in patients up to 48 h prior to enrollment. Enrollment APACHEIII (*p* = 0.016) and lactate (*p* = 0.115) values were increased in nonsurvivors (96.5 CI 82.1–111.8; 4.5 mmol/l, CI 2.3–6.9, respectably) compared to survivors (71.8 CI 51.3–92.3; 2.2 mmol/l, CI 1.2–3.2, respectively).

### Global mass spectrometry analysis

We previously demonstrated that metabolomic changes in patients with sepsis enrolled in the emergency department and the medical intensive care unit differentiated between survival and nonsurvival^8,9,24^. In this study, we sought to determine metabolomic changes in patients enrolled into the ICU with ARF. While most of these patients met the criteria for sepsis, enrollment criteria was not associated with documented sepsis. Global serum metabolomic analysis was performed using semi-quantitative mass spectrometry as previously described^8,9,24^. Metabolomic changes were measured at day 1 and day 3 as well as day of discharge in 180d survivors. The analysis identified 764 annotated metabolites. ANOVA (all pairwise comparisons, 5% false discovery rate (FDR)) found that there were significant differences between metabolic profiles on day 1 and day 3 in nonsurvivors compared to day 1 and day 3 in survivors (111 of 764, and 112 of 764 metabolites, respectively) and between day 1 and day 3 in nonsurvivors compared to discharge (237 of 764, and 265 of 764 metabolites, respectively; supplemental table 5). When comparing survivor day 1 and day 3 metabolomic values to discharge values, there were few changes; only 39 of 764 possible metabolomic differences were noted in day 1 survivors versus those who were discharged and 12 of 764 were observed in day 3 survivors versus those that were discharged (supplemental table 5).

**Figure 1:**
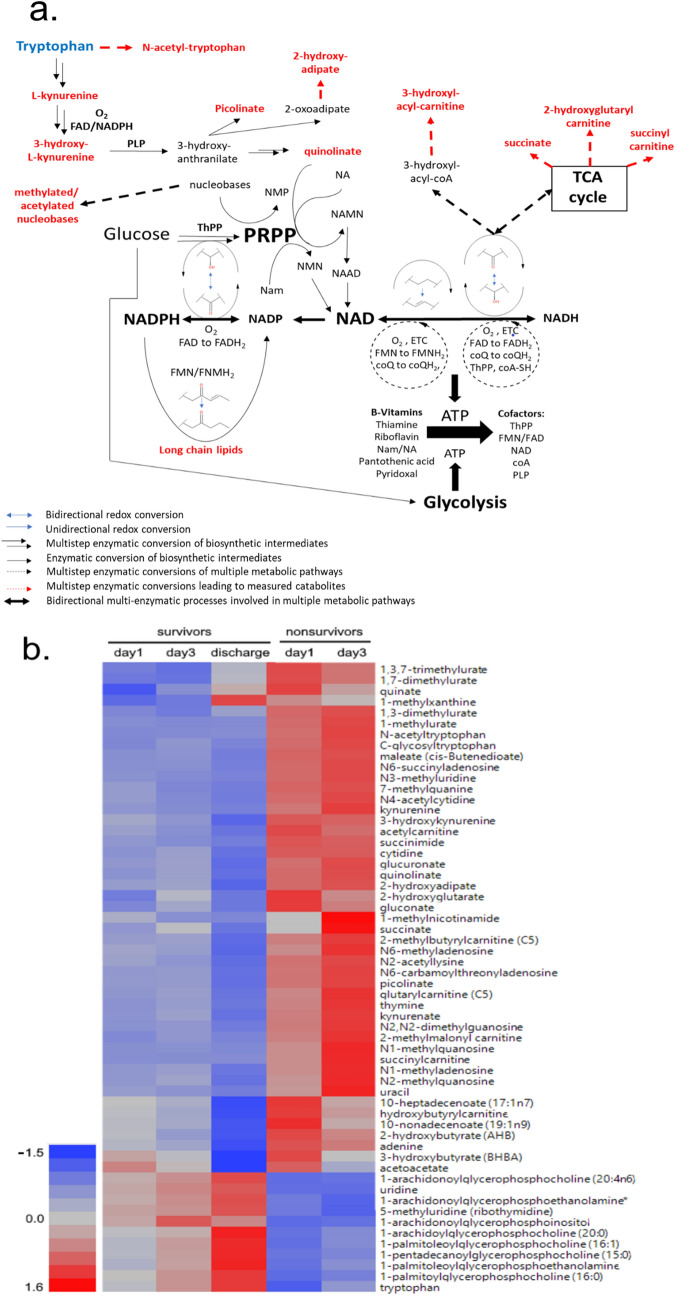
ARF leads to dysregulated NAD metabolism in nonsurvivors. (**A**) Pathway analysis of bioenergetic changes in ARF nonsurvivors. A loss of functional levels of PRPP due to a decline in the levels of functional B-vitamin derived cofactors and nucleotides can explain the accumulation of catabolites (red) observed to greatly differ between survivors and nonsurvivors. (**B**) Ward hierarchical cluster heat map of dysregulated bioenergetic metabolites in ARF nonsurvivors. Concentration of metabolites is depicted by least-squares means with red being increased concentration and blue as reduced concentration in the serum. Metabolites for presentation were selected as representatives of the primary affected pathways represented in 1A. Heatmap made utilizing JMP Genomics 8.0, https://www.jmp.com/en_us/software/genomics-data-analysis-software.html. NAD: nicotinamide adenine dehydrogenase; NAMN: nicotinic acid mononucleotide; NMN: nicotinamide mononucleotide; NAAD: nicotinic acid adenine dinucleotide; ETC: electron transport chain; TCA: tricarboxylic acid; NMP: ribonucleoside monophosphate; ThPP: thiamine pyrophosphate; FMN: flavin monophosphate; FMNH_2:_ flavin mononucleotide reduced form; FAD: flavin adenine dinucleotide; FADH_2_: flavin adenine dinucleotide reduced form; coA-SH: coenzyme-A; PLP: phosphopyridoxal; coQ: coenzyme Q; CoQH_2_: coenzyme Q, reduced form.

Among the most conspicuous metabolomic changes in survivors versus nonsurvivors were those related to the consumption and/or biosynthesis of tryptophan (de novo), nicotinic acid (NA) or nicotinamide (Nam) for NAD (Fig. 1). NAD is a key cofactor central to metabolism and mitochondrial function^25^. In addition to tryptophan, accumulation of all biosynthetic intermediates upstream of the ribosylation step of quinolinate is observed, along with that of the derived catabolite, picolinate^26^. Importantly, the levels of methylnicotinamide, a catabolite of NAD, is increased at day 3 in nonsurvivors, while these levels decline in convalescing patients. This observation is consistent with over-consumption of NAD by poly-adenosine diphosphate ribose polymerases (PARPs) and sirtuins^27–30^.

Significantly, we also noted an increase in methylated and acetylated purine and pyrimidine nucleobases. These changes point to the accumulation of materials upstream of ribosylation processes, and to a compromised pentose phosphate pathway. Together, these markers are highly predictive of mortality, and suggest that nonsurvivors have severe decrements in mitochondrial function and metabolism that may contribute to multiple organ dysfunction secondary to critical illness^3,18,31^.

**Figure 2:**
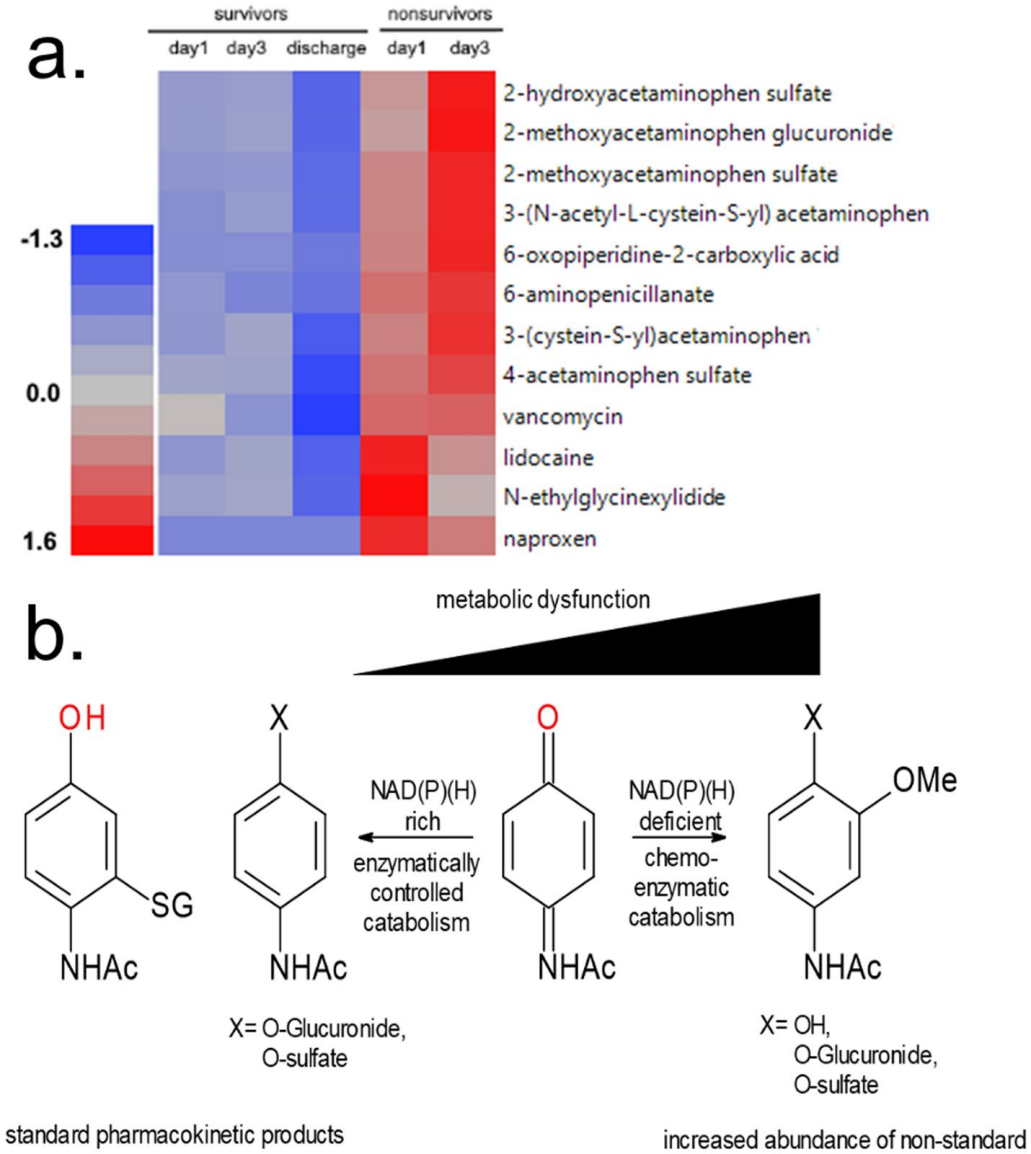
Dysregulated Metabolism of Acetaminophen. (**A**). Ward hierarchical cluster heatmap of dysregulated drug metabolism in nonsurvivors. Concentration of metabolites is depicted by least squares means with red being increased concentration and blue as reduced concentration in the serum. Heatmap made utilizing JMP Genomics 8.0, https://www.jmp.com/en_us/software/genomics-data-analysis-software.html. (**B**) Accumulation of 2-methoxyacetaminophen derivatives are evidence of an overwhelmed NQO1/NQO2 redox process and accumulation of the quinone intermediate. As glutathione levels decline, methyl-derivatives accumulate, likely derived from the nucleophilic addition of water on the quinone intermediate followed by methylation. *N*-acetyl cysteine and acetyl cysteine conjugated to the quinone intermediate and the resulting adducts, cysteine-derivatives of acetaminophen accumulate in nonsurvivors. Metabolites selected are representative of the significantly different drug xenobiotics identified by the semiquantitative analysis.

An acute and sustained imbalance of NAD cofactors can also impact catabolism of multiple drugs and xenobiotics with one well-known example being acetaminophen. Glutathione, at the heart of one of the major oxygen radical detoxifying pathways, is a major partner in the detoxification of acetaminophen, and some catabolites of acetaminophen become markers of glutathione depletion^32,33^. Critically, maintenance of glutathione levels requires its effective recycling processes that use the reduced form of NADP, the phosphorylated form of NAD. In this study we found significant differences in the profile of six acetaminophen-related catabolites in nonsurvivors compared to survivors (Fig. 2). These metabolites do not normally occur in healthy catabolism.

**Figure 3:**
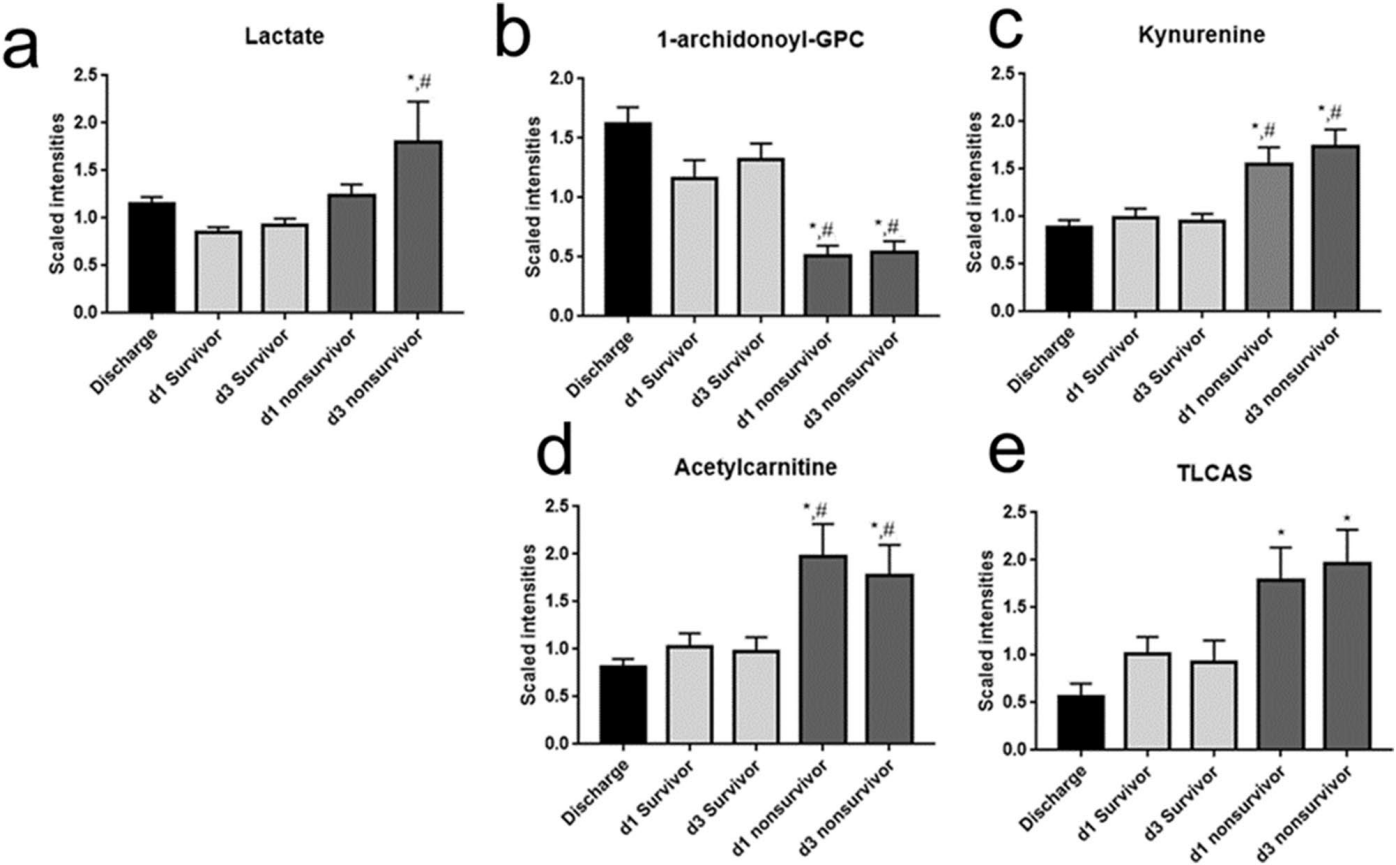
Demographics and semiquantitative analysis of sepsis outcomes predictive metabolite. (**A**–**F**) semiquantitative analysis of outcome predictive metabolites. Significant difference using ANOVA and 5% FDR. *, significantly different from discharge; #, significantly different from time-matched survivor. Figures made utilizing GraphPad Prism 7.0. https://www.graphpad.com/scientific-software/prism/.

Semiquantitative data demonstrated that lactate was moderately, but significantly increased in nonsurvivors only at day 3 (Fig. 3a). Consistent with previous findings in sepsis nonsurvivors, we detected increased acylcarnitines, bile acids, sulfated steroids, modified nucleosides, and decreased acyl-GPCs (supplemental table 5). The concentration of 1-archidonoyl-GPC was significantly reduced on day 1 and day 3 in nonsurvivors compared to survivors at discharge, while acetylcarnitine, kynurenine and TLCAS were significantly increased compared to discharge (Fig. 3b–e). Ketone bodies, 3-hydroxybutyrate (BHBA) and acetoacetate, were increased on day 1 and day 3 for survivors compared to discharge, while fructose was decreased on day 1 and day 3 in survivors compared to discharge. The increase in ketone bodies suggests increased bioenergetic stress during critical illness or use of alternative precursors (amino acids) to mitochondrial acetyl-CoA. Ketone bodies can also be produced by the reduction of the carbonyl groups to regenerate NAD from NADH^34^.

### Quantitative targeted assay analysis of metabolomic changes

In an NHP model of sepsis, we previously showed that four metabolomic pathways strongly predict patient outcomes due to sepsis and were validated in human studies^9,18^. Accordingly, we hypothesized that a MetSeP score utilizing representative metabolites of the four metabolomic pathways previously identified in human and NHP sepsis studies would similarly predict ARF patient outcomes in the MICU with high accuracy. Selection of the representative metabolites was determined based on whether they were also identified in the quantitative targeted assay analysis. Two commercially available kits were used to quantify over 200 biomarkers including representative metabolites from the four biochemical pathways of interest.

**Figure 4:**
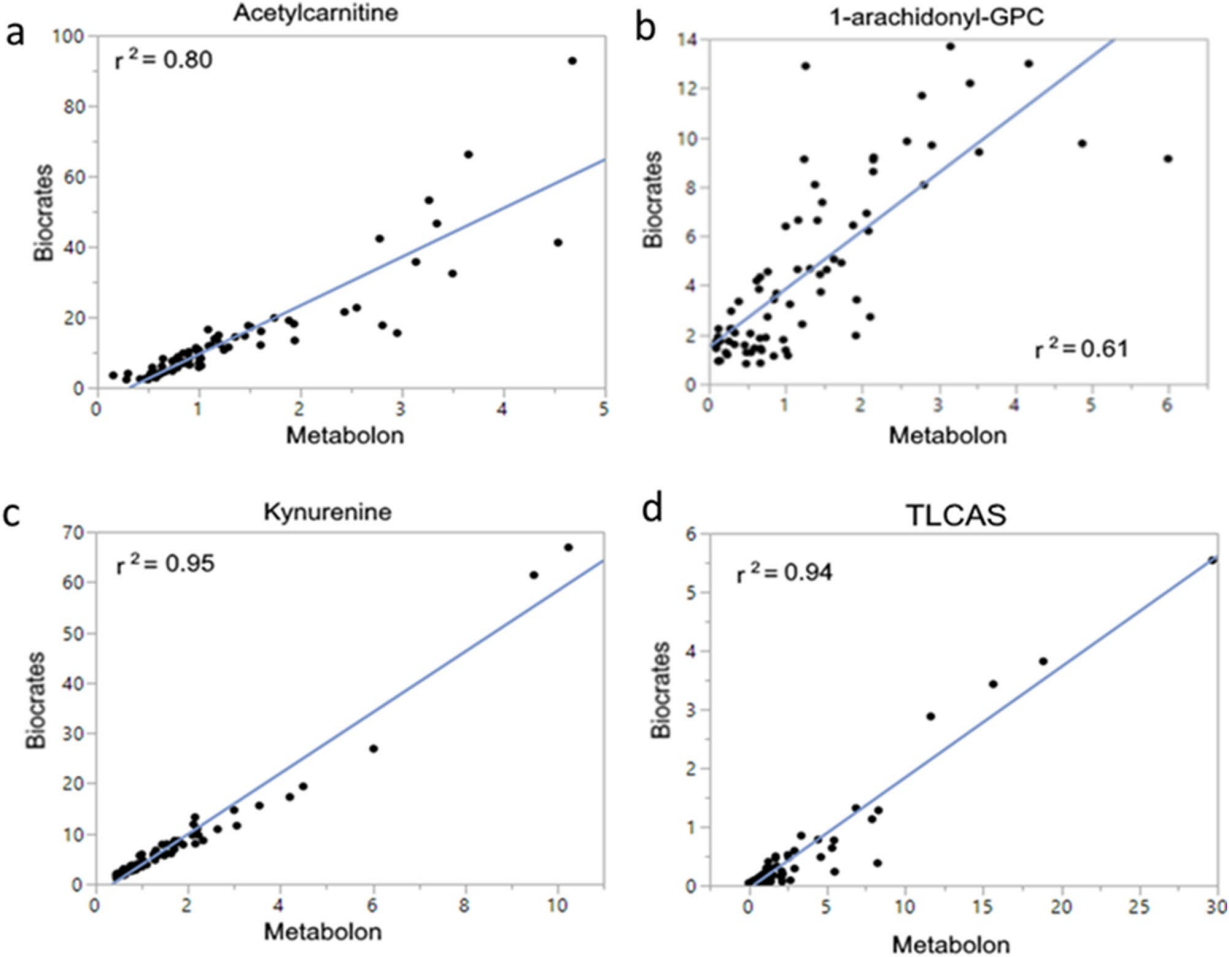
Correlation analysis of quantitative and semiquantitative results. Spearman’s Rank correlation analysis performed on quantitative (Biocrates) and semiquantitative (Metabolon) results shows a strong correlation (r = 0.79–0.97) and comparable predictive value for each pathway measured. Figures made utilizing JMP Genomics 8.0, https://www.jmp.com/en_us/software/genomics-data-analysis-software.html.

Seventy-five serum samples were tested using these commercial kits for direct comparison to the semiquantitative results. The analysis determined that 75 metabolites could be measured within the kits’ dynamic range. ANOVA (5% FDR) detected 29 metabolites that were significantly different in at least one comparison of nonsurvivors versus survivors (Table 2). We noted increased concentrations of kynurenine derivatives, acylcarnitines and conjugated bile acids as well as decreased concentrations of acyl-GPCs were significantly different between nonsurvivors and survivors. Spearman’s rank correlation analysis of semiquantitative versus quantitative results was high, with r between 0.79 and 0.97 (Fig. 4a–d).

**Table 2:** Quantitative targeted assay analysis.

	Discharge	Survivor		Nonsurvivor	
		Day1	Day3	Day1	Day3
*Amino acids*					
Alanine	317 ± 26.8	195 ± 20.2*	209 ± 20.1	218 ± 25.0*	516 ± 210
Arginine	91.6 ± 10.0	73.9 ± 10.3	76.9 ± 9.4	55.4 ± 9.2*	55.7 ± 9.8*
Phenylalanine	81.4 ± 6.8	120.8 ± 24.8	103.9 ± 18.6	120.4 ± 14.7	185.7 ± 35.4*,#
Glycine	333.8 ± 22.6	233.2 ± 19.5*	236.6 ± 24.3	202.5 ± 11.9*	301.7 ± 71.3
Proline	194.7 ± 15.2	169.8 ± 18.4	165.7 ± 16.6	181.5 ± 24.6	290.6 ± 45.8#
Serine	111.0 ± 7.4	85.0 ± 7.4	94.6 ± 8.5	59.3 ± 3.7*	77.1 ± 8.5*
Threonine	155.4 ± 12.6	104.9 ± 11.9*	125.3 ± 17.5	92.3 ± 9.2*	152.3 ± 35.1
*AA Derivatives*					
Asymmetric dimethylarginine	0.61 ± 0.03	0.61 ± 0.06	0.55 ± 0.04	0.71 ± 0.1	0.84 ± 0.08#
Creatinine	161.4 ± 48.7	221.4 ± 75.2	211.7 ± 69.9	293.9 ± 47.5	339.0 ± 47.2*,#
Sarcosine	7.7 ± 0.7	5.1 ± 0.5*	5.3 ± 0.4	5.3 ± 0.6*	6.2 ± 0.7
t4-OH-Pro	14.5 ± 1.8	12.9 ± 2.9	8.4 ± 1.1	15.1 ± 3.0	20.9 ± 4.0#
*NAD*+					
Tryptophan	58.2 ± 5.0	42.6 ± 4.2	47.7 ± 5.7	31.0 ± 3.4*	39.0 ± 5.2
Kynurenine	3.5 ± 0.4	4.7 ± 0.8	4.0 ± 0.6	11.7 ± 4.1*	14.0 ± 3.8*,#
*Carnitine esters*					
Acetylcarnitine	7.0 ± 0.7	9.9 ± 1.5	9.1 ± 1.6	23.6 ± 6.5*	19.6 ± 4.4*
C3-DC (C4-OH)	0.14 ± 0.01	0.19 ± 0.03	0.16 ± 0.02	0.33 ± 0.1*	0.30 ± 0.05*,#
C6 (C4:1-DC)	0.09 ± 0.00	0.12 ± 0.02	0.12 ± 0.02	0.23 ± 0.1*	0.25 ± 0.05*,#
Octanoylcarnitine	0.18 ± 0.01	0.19 ± 0.02	0.21 ± 0.03	0.32 ± 0.1	0.35 ± 0.06*
*Acyl-GPCs*					
lysoPC a C16:0	74.2 ± 7.5	34.4 ± 4.0*	48.0 ± 6.8	16.4 ± 2.4*,#	15.5 ± 3.4*,#
lysoPC a C16:1	2.62 ± 0.30	1.07 ± 0.10*	1.53 ± 0.21*	0.58 ± 0.1*,#	0.61 ± 0.12*,#
lysoPC a C17:0	1.37 ± 0.15	0.63 ± 0.06*	0.87 ± 0.12	0.42 ± 0.0*	0.41 ± 0.08*,#
lysoPC a C18:0	20.2 ± 2.4	9.8 ± 1.4*	13.4 ± 2.1	4.9 ± 0.6*,#	5.3 ± 1.5*,#
lysoPC a C18:1	21.0 ± 4.3	8.8 ± 0.9*	13.3 ± 2.0	4.3 ± 0.5*,#	5.1 ± 1.2*,#
lysoPC a C18:2	29.9 ± 4.3	10.8 ± 1.6*	17.5 ± 3.3	4.8 ± 0.5*,#	5.6 ± 1.7*,#
lysoPC a C20:3	2.49 ± 0.42	1.23 ± 0.11*	1.48 ± 0.19	0.92 ± 0.1*	0.98 ± 0.18*
lysoPC a C20:4	8.7 ± 0.9	4.2 ± 0.5*	5.5 ± 0.9	2.1 ± 0.2*,#	2.1 ± 0.5*,#
*Phosphatidyl-cholines*					
PC aa C32:0	16.7 ± 2.4	15.9 ± 1.9	15.8 ± 1.6	32.5 ± 8.6	50.5 ± 25.6#
PC ae C30:0	0.40 ± 0.03	0.44 ± 0.05	0.40 ± 0.03	0.69 ± 0.1	0.81 ± 0.14*,#
PC ae C40:1	0.98 ± 0.15	0.55 ± 0.05*	0.72 ± 0.11	0.46 ± 0.1*	0.57 ± 0.21
*Bile acids*					
Taurolithocholic acid sulfate	0.09 ± 0.01	0.21 ± 0.04	0.23 ± 0.09	0.71 ± 0.4	0.90 ± 0.34*

Metabolites were measured in nonsurvivors and survivor patients at day1 and day 3 of enrollment and in survivor patients at discharge using targeted assays. Significant difference using ANOVA and 5% FDR. *, significantly different from discharge; #, significantly different from time-matched survivor. Results are presented as μM mean ± standard error of mean.

### Predictive modeling of semiquantitative and quantitative results demonstrates the MetSeP score has improved 28d outcomes prediction compared to APACHEIII

**Figure 5:**
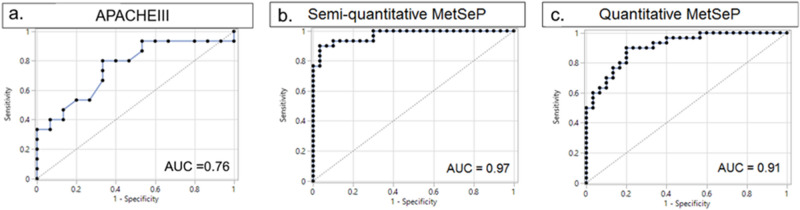
AUROC analysis of lactate and MetSeP score. APACHEIII was measured in the TARGET patients at ICU admittance. MetSeP was measured using semiquantitative results from Metabolon and quantitative analyses. Logistic regression shows that APACHEIII values were less accurate (area under the curve; AUC = 0.76) for patient outcome prediction than MetSeP scores (AUC = 0.97 and 0.91, respectively). Figures made utilizing JMP Genomics 8.0, https://www.jmp.com/en_us/software/genomics-data-analysis-software.html.

To determine the performance of the MetSeP score, logistic regression analysis of APACHEIII measurements in the TARGET cohorts was compared to outcomes prediction of the MetSeP score in both semiquantitative and quantitative datasets. The APACHEIII values were moderately accurate for prediction of patient outcomes (AUROC = 0.76; Fig. 5a). Logistic regression analysis of the composite changes in 1-arachidonyl-GPC, acetylcarnitine, kynurenine and TLCAS in the semiquantitative datasets was able to predict outcomes with greater accuracy (AUC = 0.97; Fig. 5b). The MetSeP score was recalculated in these samples also using logistic regression analysis of the composite changes to quantitative results of kynurenine, 1-arachidonyl-GPC, acetylcarnitine, and TLCAS; the quantitative results also predicted patient outcomes with high accuracy (AUC = 0.91; Fig. 5c).

## Discussion

There is generally poor understanding of how biomarkers in sepsis and ARF are related to mechanisms and pathophysiology^35^; moreover, the capacity of sepsis and ARF to lead to morbidities and mortalities^36^, increased hospital stays, and persistent decrements in quality of life in discharge patients^37^, demonstrate a need for biomarkers that are more reliable than conventionally used ordinal or other scoring systems of disease severity such as APACHEIII or lactate.

We previously developed a clinico-metabolomic model that could predict the patient outcomes of patients enrolled in the ED and MICU better than lactate, APACHEII (an older calculation of the APACHEIII score) and systemic organ failure assessment (SOFA) in patients with sepsis on the basis of at least two systemic inflammatory responses and suspected infection^8^. Subsequently we performed a similar study in an NHP model of sepsis integrating metabolomic and lung transcriptomic changes which enabled identification of four biochemical pathways that predicted sepsis diagnosis and poor outcomes^9^, thus providing insight into biologically relevant pathways and retrospectively validating the earlier results in human patients.

The pathways delineated by our experiment in the NHP model were related to platelet activating factors^8,11,12^, liver cholestasis bile acids^13^, NAD biosynthesis^14^ and acylcarnitines, the latter two suggestive of dysregulated β-oxidation of the TCA cycle^8^.

In the current study, broad spectrum, semiquantitative analysis of the metabolomic changes in patient serum found that more than 230 metabolites were significantly different in nonsurvivors relative to discharged patient samples, and approximately 110 biomarkers differed between nonsurvivors and survivors in a time-matched analysis. Consistent with findings previously reported by us and others, we found significant increases in acylcarnitines, modified nucleosides, kynurenine-related catabolites, sulfated steroids, bile acids, and decreased concentrations of acyl-GPCs^8–10,15,38,39^. An aim of this study was to develop outcome markers of critical illness that are pathophysiologically relevant. Along these lines, one of the most consistent metabolic pathways altered in our ARF cohort was the pentose phosphate-dependent production of NAD, specifically the de novo NAD biosynthetic pathway. To be functional, NAD requires contributions from other key enzyme cofactors, including its phosphorylated form, NADP, and cofactors derived from thiamine, riboflavin, pantothenic acid, and pyridoxal. Furthermore, the metabolic imbalance noted in ARF nonsurvivors appears to be established and irreversible as evidenced by the accumulation of TCA cycle and lipid metabolites (acylcarnitines). In light of these consistent metabolomic changes, we propose that mortality associated with ARF is driven by depletion of the NAD pool and ultimately ATP levels caused in part by a disrupted pentose phosphate pathway and reduced levels of phosphoribosyl pyrophosphate (PRPP) that limit conversion of nucleobases, nicotinic acid, and nicotinamide to their nucleotide monophosphate form.

Dysregulated NAD metabolism also could impact the disposition and effect of therapeutic agents and endogenous mediators of ARF patients, the latter including PARP1, sirtuins, glutathione, and others^40–45^. In this context, we noted in nonsurvivors a striking increase in methoxy-acetaminophen-related catabolites. These catabolites greatly differ from glucuronate, sulfate or glutathione-conjugates, which are main catabolites of acetaminophen in classical pharmacokinetic studies^46^. This observation indicates insufficient glutathione in nonsurvivors (Fig. 3). Critically, maintenance of glutathione levels is directly dependent on the availability of NADPH, which is most effectively produced from NADP during the production of PRPP.

Recent metabolomics studies of human plasma have identified the kynurenine pathway, acylcarnitines and acyl-GPCs as prognostic markers of Covid-19 disease severity^47–50^. A consistent message is that metabolic dysfunction occurs in respiratory distress. Yet, the targeted nature of the metabolites being detected limits our understanding of the underpinning mechanisms promoting the observed metabolic outcomes. Here, we demonstrated that the metabolic shifts that have been identified and correlated with ARDS outcomes could be rationalized for their impact on drug metabolism. This is exemplified by the observed metabolism of acetaminophen that recapitulates at least one mechanism of dysfunction, which is directly related to cellular metabolism and redox cofactors. Viewed collectively, these observations point to the prospect that the metabolic status of a patient could alter the course of ARF by impacting the metabolism of both drugs and endogenous regulators of disease. Obviously, this concept needs to be explored by studies of a design different that that used herein.

The present observations suggest that the risk of death in ARF patients could be stratified using a metabolomic analysis focused on NAD-related pathways. From a mechanistic perspective, targeted metabolomics of hospitalized patients with ARF also could differentiate between bioenergetic profiles, with one endotype assigned to patients with normal NAD metabolism and another applied to patients with critical metabolic dysfunction centred upon dysregulated NAD-related pathways. Such a distinction could be important for optimizing a nutritional regimen of pre-ribosylated precursors to NAD. In this context, administration of precursors to NAD—nicotinamide ribose (NR) or NMN, especially if combined with thiamine supplementation-might offer means of early remediation that could “kick start” NAD and ATP-generation in some patients exhibiting an NAD deficient metabolic profile^51–53^. NRH could also prove a suitable precursor^52,54^, but the pharmacological properties and toxicity profile of this particular NAD precursor remain to be established before it can be considered for human use.

Against this background, we wanted to determine if metabolomics biomarkers would predict patient outcomes due to ARF. Moreover, ideal biomarkers should be linked to the pathophysiology driving patient outcomes and have utility in selecting pharmacologic interventions. Finally, it is of practical importance that the metabolomic biomarkers can be quantified using targeted MS in a relatively simple and preferably a commercially available kit.

Based on the above results, we developed a MetSeP score that encompassed four representative metabolites that could be measured in a commercially available kit. Logistic regression was used to calculate the MetSeP score utilizing measured values of TLCAS, acetylcarnitine, kynurenine and arachidonoyl-GPC comparing patient survival and nonsurvival. The semiquantitative results predicted patient outcomes with an exceptionally high AUROC = 0.97 and the semi-quantitative (broad-spectrum MS) and quantitative (targeted MS) values were highly correlated (r^2^ = 0.79–0.97). Furthermore, calculation of the MetSeP score utilizing quantitative data was approximately as accurate as the semiquantitative data with an AUROC = 0.91. While APACHEIII scores were statistically different in nonsurvivors from survivors (Table 1), a logistic regression analysis of APACHEIII did not predict outcomes as well as the MetSeP scores, as indicated by an AUROC = 0.76. Collectively, these findings indicate that metabolomic changes, measured quantitatively using commercially available kits on UHPLC MS platforms, predict outcomes better than APACHEIII. Optimization of these assays could thus provide results quickly and predict patient outcomes with high accuracy. However, a large, multisite trial is needed to validate this prospectively.

This study has certain limitations. While most of the patients were assumed to have developed sepsis, enrollment was not based on infection status but rather development of ARF and requirement for ventilation. Sepsis is a common cause of ARF; however, other conditions such as inflammatory pneumonias and shock may lead to ARF and thus treatment with mechanical ventilation^36^. Moreover, although most of the patients were treated with broad spectrum antibiotics, this does not exclude the possibility that some of the patients were not infected. Therefore, the markers may be nonspecific critical illness markers rather than unique to sepsis or ARF. Additionally, the study population was small, and patient selection is potentially biased as nonsurvivors were preferentially selected in this nested case–control study. Due to the limited size of the study, multivariate analysis of factors such as age, race, sex or APACHEIII score was not performed. Finally, while the study allowed for inclusion of noninvasive ventilation, all of the enrolled patients were mechanically ventilated. It may be interesting in future studies to determine how modern therapies may affect the metabolome. Despite these limitations, the present findings confirmed that metabolomic changes were similar to what has been previously reported^8–10^, and to this, add an independent validation of the MetSeP score in a unique cohort with a diverse study population. Further, the selected metabolites were intentionally limited to those that could be quantitatively measured using a commercial assay. NAD-pathway specific metabolites and catabolites or others more predictive than those selected for this study could be worthwhile since modulation of such entities is potentially actionable and offer the possibility of monitoring positive physiological impact.

In conclusion, collectively, the MetSeP score represents a metabolomic endotype, defined as a subgroup within a patient population that can be distinguished by a shared disease process^55^. Moreover, the pathophysiologic features of these biomarkers have the potential to direct new therapies that target immune dysregulation and bioenergetic insufficiency^16^.

## Supplementary Information

Supplementary Information.
